# Integrating socio-psychological factors in the SEIR model optimized by a genetic algorithm for COVID-19 trend analysis

**DOI:** 10.1038/s41598-024-66968-0

**Published:** 2024-07-08

**Authors:** Haonan Wang, Danhong Wu, Jie Luo, Junhui Zhang

**Affiliations:** 1 School of Public Health, Southwest Medical University, No. 1, Section 1, Xianglin Road, Longmatan District, Luzhou, 646000 Sichuan People’s Republic of China; 2 Department of Applied Mathematics, Chengdu University of Information Technology, Chengdu, 610225 Sichuan People’s Republic of China; 3 Department of Psychology, Durham University, Durham, DH1 3LE UK

**Keywords:** COVID-19, Dynamic model, GA-SEIR model, Epidemic forecasting, Socio-psychological factors, Optimization algorithm, Epidemiology, Preventive medicine, Applied mathematics, Statistics

## Abstract

The global spread of COVID-19 has profoundly affected health and economies, highlighting the need for precise epidemic trend predictions for effective interventions. In this study, we used infectious disease models to simulate and predict the trajectory of COVID-19. An SEIR (susceptible, exposed, infected, removed) model was established using Wuhan data to reflect the pandemic. We then trained a genetic algorithm-based SEIR (GA-SEIR) model using data from a specific U.S. region and focused on individual susceptibility and infection dynamics. By integrating socio-psychological factors, we achieved a significant enhancement to the GA-SEIR model, leading to the development of an optimized version. This refined GA-SEIR model significantly improved our ability to simulate the spread and control of the epidemic and to effectively track trends. Remarkably, it successfully predicted the resurgence of COVID-19 in mainland China in April 2023, demonstrating its robustness and reliability. The refined GA-SEIR model provides crucial insights for public health authorities, enabling them to design and implement proactive strategies for outbreak containment and mitigation. Its substantial contributions to epidemic modelling and public health planning are invaluable, particularly in managing and controlling respiratory infectious diseases such as COVID-19.

## Introduction

The outbreak of coronavirus disease 2019 (COVID-19), caused by the novel severe acute respiratory syndrome coronavirus 2 (SARS-CoV-2), first reported in Wuhan, Hubei Province, China, in early December 2019, rapidly escalated into an unprecedented global health crisis^1^. Recognized by the International Committee on Classification of Viruses in February 2020, the rapid spread of the pandemic highlighted the profound need for advanced research in respiratory infectious disease modelling. This urgent situation necessitated robust, adaptable models that can address the current challenges and prepare for future outbreaks caused by different pathogens.

The modelling of the COVID-19 pandemic has experienced a surge in research, with the susceptible-exposed-infected-removed (SEIR) model and its variants being widely utilized to simulate and predict the course of the outbreak. Although these models theoretically provide a framework for understanding the dynamics of the disease, they have faced significant challenges in practical application. Early applications of the SEIR model, such as those by Hao et al.^2^, provided initial insights into the virus's spread in Wuhan but significantly overestimated the actual case numbers due to an incomplete understanding of the virus's transmissibility and the effectiveness of initial public health responses.

In refining these approaches, Wu et al.^3^ tailored the SEIR model to better fit the COVID-19 context in China, incorporating adjustments for asymptomatic transmission and evolving governmental policies but still underestimating the overall impact of the outbreak. Similarly, Yang et al.'s comprehensive epidemiological approach integrated factors such as human mobility and public health measures to predict both domestic and international spread from Wuhan^4^. Despite these enhancements, the model struggled with the accurate prediction of the pandemic’s scale and intensity, highlighting the virus's mutability and the diversity of global responses.

With these evolving challenges, extensive research from various perspectives has enriched the data and theoretical frameworks available. These studies have deepened our understanding of the pandemic and highlighted the complexities of epidemic modelling. However, existing models, including those discussed above, still face significant limitations in terms of prediction accuracy and practical application, emphasizing the need for further methodological innovations.

Recent advancements in the SEIR model have been significantly driven by the introduction of a genetic algorithm (GA) for parameter optimization, a key contribution to the evolution of epidemic forecasting. In addition to GA, factors such as the incubation period and the isolated population have also been incorporated, further enhancing these models. This approach, as exemplified in the works of Qiu et al.^5^, has enabled more precise predictions of epidemic trends and the attainment of accurate epidemic-related parameters. However, while these improved SEIR models, including the GA-SEIR version, have made substantial progress, they still tend to oversimplify the multifaceted nature of real-world scenarios. This results in certain limitations in their broader predictive capabilities, as they may not fully account for the complex interplay of the standard SEIR model’s parameters present in actual epidemic situations.

Considering the literature that highlights the significant role of human behavior and social responses influenced by socio-psychological factors in the spread of respiratory infectious diseases, our study integrates these factors into the SEIR (GA-SEIR) model. This enhancement aims to improve the model's predictive accuracy for the spread of COVID-19 and provide a comprehensive understanding of how social behaviors influence disease transmission and control. Despite a reduction in widespread testing and a flattened incidence curve in the current state of the COVID-19 pandemic, the need for advanced modelling remains critical. It emphasizes the importance of preparedness for future infectious disease outbreaks, particularly in grasping the complex interplay between epidemiological dynamics and social-psychosocial factors^6^. This refined GA-SEIR model not only aids in the formulation of effective strategies for managing epidemic progression but also serves as a valuable scientific foundation for global policymakers in handling similar respiratory infectious diseases, such as COVID-19^7^.

## Methods

### Data source

This study involved the creation of a comprehensive SEIR model analysis database employing three distinct datasets. The first dataset, sourced from the Wuhan Municipal Health Commission's official website (http://wjw.wuhan.gov.cn), included daily counts of individuals in each SEIR category (susceptible, exposed, infected, and removed) in Wuhan City. These data were used exclusively for constructing the SEIR model and determining its parameters.

The second dataset, obtained from Johns Hopkins University's COVID-19 section (https://coronavirus.jhu.edu), involved daily counts of individuals in each SEIR category, average infection rates, and associated socio-psychological factors—compliance and sentiment—for a specific period and region in the United States. These two factors, compliance and sentiment, directly influence the model's key parameters: the transmission rate ($\alpha$) and the removed rate $(\delta$). This illustrates how changes in public behavior significantly impact the progression of the epidemic. This dataset was used to construct and iteratively refine the GA-SEIR model, with socio-psychological factors being incorporated for a detailed comparative analysis between the traditional and enhanced models^8^.

For external validation, we included a third dataset focusing on the COVID-19 situation in mainland China as of April 2023. This dataset, obtained from the National Health Commission of the People's Republic of China (http://www.nhc.gov.cn), played a crucial role in validating the accuracy and applicability of the refined GA-SEIR model. By applying our models to these current and region-specific data, we were able to confirm their effectiveness and relevance in the context of evolving pandemic trends.

### SEIR model

#### Data preparation and theoretical basis of the standard SEIR model

**Figure 1:**
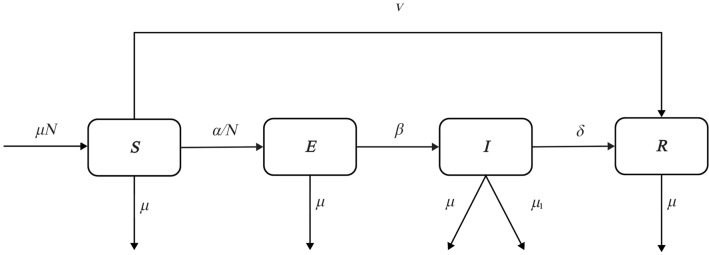
Standard SEIR model for COVID-19 transmission: illustrating population dynamics in susceptible (*S*), exposed (*E*), infectious (*I*), and removed (*R*) groups and periodic parameter comparisons during the epidemic. *Note*: *S* represents healthy individuals who have not been infected with the virus and lack immunity; *E* denotes individuals who are in the incubation period of infection after effective contact with the infected patient; *I* indicates individuals who are symptomatic and infectious; *R* includes individuals who have either recovered from or passed away due to the disease; *N* denotes the total population of the city is kept constant; $\alpha$ denotes the effective contact rate between susceptible persons and infected patients; $\beta$ indicates the probability of the exposed group becoming infected; $\mu$ represents the natural mortality rate affecting all population groups, reflecting non-disease-specific deaths; ${\mu }_{1}$ specifically denotes the mortality rate due to COVID-19, affecting the transition from *I* to *R*; $\nu$ indicates the vaccination rate of the susceptible group; and $\delta$ represents the cure rate.

First, we set the time series to span 150 days, with daily COVID-19 data collected in Wuhan City, China. Each day, a health status assessment is conducted for all individuals considering their state from the previous day^9^. The standard SEIR model divides health status into four defined population groups: *S* (susceptible), *I* (infective), *E* (exposed) and *R* (removed)^10^. The model determined that the population was evenly mixed, without special isolation or the implementation of relevant policies^11^. The transfer process of COVID-19 transmission and the parameters used in this study are shown in Fig. 1. $S\left(t\right)$ denotes healthy individuals who have not been infected with the virus and lack immunity, $E\left(t\right)$ denotes individuals who are in the incubation period of infection after effective contact with the infected patient, $I\left(t\right)$ denotes individuals who are symptomatic and infectious, and $R(t)$ denotes individuals who have either recovered from or passed away due to the disease^12^.

$S\left(t\right),E\left(t\right),I\left(t\right),R\left(t\right)$ represents the total number of individuals in each group at time *t*, respectively. In addition, the total population of the city is kept constant as ${\rm N}$, and special circumstances such as population flow are not considered, which is denoted as $S\left(t\right)+E\left(t\right)+I\left(t\right)+R\left(t\right)=N$ ^13^.

For group interactions, we set the effective contact rate between susceptible persons and infected patients as $\alpha$. The probability of the exposed group becoming infected is represented by $\beta$, which is the reciprocal of the incubation period days. The cure rate, $\delta$, indicates the probability of removed of the infected group, assuming no reinfection of pathogens after recovery^14^. The mortality rate affecting the transition of individuals from the infectious group $I(t)$ to the recovered group $R(t)$ due to COVID-19 is represented by ${\mu }_{1}$, which specifically describes the rate at which symptomatic and infectious individuals die from the disease. Finally,$\nu$ is the vaccination rate of the susceptible group, with vaccinated individuals transitioning from $E(t)$ to $R(t),$ assuming natural mortality affects all compartments, represented by $\mu$, impacting the general mortality rate not directly related to the disease^15,16^.

The details within Fig. 1 are expressed by specific differential equations as follows^17^:

$$\frac{dS\left(t\right)}{dt}=\mu N-\frac{\alpha IS}{N}-\mu S-\nu S$$

$$\frac{dE\left(t\right)}{dt}=\frac{\alpha IS}{N}-\beta E-\mu E$$

$$\frac{dI\left(t\right)}{dt}=\beta E-{\mu }_{1}I-\delta I-\mu I$$

1 $$\begin{array}{c}\frac{dR\left(t\right)}{dt}=\delta I+{\mu }_{1}I+\nu S-\mu R\end{array}$$

#### Determine the transmission equilibrium point

On the initial day (day zero) of transmission, the system is considered to be in equilibrium, denoting that the rate of change of the number of each group (*S, E, I, R*) over time is zero; in other words, their numbers remain constant. To find the equilibrium point, we set the left side of the differential equations of the model to zero, which yields four Eqs. ^18^.

At this point, we normalize the population groups by the total population (*N*) using the formula $S=\frac{S}{N},E=\frac{E}{N},I=\frac{I}{N},R=\frac{R}{N}$, and we obtain Eq. (2):

$$\mu -\alpha IS-\mu S-\nu S=0$$

$$\alpha IS-\beta E-\mu E=0$$

$$\beta E-{\mu }_{1}I-\delta I-\mu I=0$$

2 $$\begin{array}{c}\delta I+{\mu }_{1}I+\nu S-\mu R=0\end{array}$$

Since both day zero and the day of epidemic elimination are at the disease-free equilibrium point^18^, the *E* and *I* values are zero at these times. These values are then substituted into Eq. (2) for calculation, and we obtain Eq. (3):

$$\mu -\mu S-\nu S=0$$

3 $$\begin{array}{c}\nu S-\mu R=0\end{array}$$

This leads us to Eq. (4):

4 $$\begin{array}{c}S=R=\frac{\mu }{\mu +\nu }\end{array}$$

At the virus equilibrium point of the COVID-19 epidemic, the transmission stabilized and reached a dynamic equilibrium state, where the population sizes of each group remained constant. This implies that the change rate of the number of individuals over time is zero^7^, as shown in Eq. (5):

$${S}_{e}=\frac{\left(\beta +\mu \right)\left({\mu }_{1}+\delta +\mu \right)}{\alpha \beta }$$

$${E}_{e}=\frac{\alpha \beta \mu -\left(\beta +\mu \right)\left({\mu }_{1}+\delta +\mu \right)}{\alpha \beta \left(\beta +\mu \right)}$$

$${I}_{e}=\frac{\alpha \beta \mu -\left(\beta +\mu \right)\left({\mu }_{1}+\delta +\mu \right)\left(\nu +\mu \right)}{\alpha \left(\beta +\mu \right)\left({\mu }_{1}+\delta +\mu \right)}$$

5 $$\begin{array}{c}{R}_{e}=\frac{\alpha {\beta }^{2}\mu \left({\mu }_{1}+\delta \right)-\beta \left(\beta +\mu \right)\left({\mu }_{1}+\delta \right)\left({\mu }_{1}+\delta +\mu \right)\left(\nu +\mu \right)+\nu {\left(\beta +\mu \right)}^{2}}{\alpha \beta \mu \left(\beta +\mu \right)\left({\mu }_{1}+\delta +\mu \right)}\end{array}$$

Therefore, the equilibrium point ${K}_{0}$ of COVID-19 is represented by the values $\left({S}_{e},{E}_{e},{I}_{e}{, R}_{e}\right)$.

#### Assessing the stability of the equilibrium point

To understand the spread of an epidemic and predict its further development with greater accuracy^20^, we need to assess the stability of the equilibrium point in both situations.

#### Disease-free equilibrium

For the disease-free equilibrium point ${K}_{0}$ observed on day zero of the outbreak, we bring it into $Jacobian$ to obtain $J\left({K}_{0}\right)$^21^ to determine its stability. The stability is confirmed by the eigenvalues derived from the characteristic equation under the conditions $S=\frac{\mu }{\mu +\nu }, I=0$ in the model, yielding the characteristic Eq. ^22^. The results for the four eigenvalues are expressed in Eq. (6):

$${\lambda }_{1}=-\left(\mu +\nu \right),{\lambda }_{2}=-\mu$$

$${\lambda }_{3}=-\frac{\left(\mu +\beta \right)}{2}-\frac{\left({\mu }_{1}+\delta +\mu \right)}{2}-\frac{1}{2}{\left({\left(\left(\mu +\delta +\mu \right)-\left(\mu +\beta \right)\right)}^{2}+\frac{4\alpha \beta \mu }{\left(\mu +\nu \right)}\right)}^\frac{1}{2}$$

6 $$\begin{array}{c}{\lambda }_{4}=-\frac{\left(\mu +\beta \right)}{2}-\frac{\left(\mu +\delta +\mu \right)}{2}-\frac{1}{2}{\left({\left(\left(\mu +\delta +\mu \right)-\left(\mu +\beta \right)\right)}^{2}+\frac{4\alpha \beta \mu }{\left(\mu +\nu \right)}\right)}^\frac{1}{2}\end{array}$$

When ${\lambda }_{1}<0,{ \lambda }_{2}<0,{ \lambda }_{3}<0$ *and* ${\lambda }_{4}<0$, this equilibrium is stable, suggesting a lack of disease spread under current conditions.

Considering the above information, $\frac{\alpha \beta \mu }{\left(\beta +\mu \right)\left({\mu }_{1}+\delta +\mu \right)\left(\mu +\nu \right)}<1, {\lambda }_{4}<0$ is the condition for the stability of equilibrium point^23^.

#### COVID-19’s equilibrium point

Following a similar methodology as above, the equilibrium point during the epidemic spread is brought in, and the eigenvalues of its matrix are calculated^24^.

After a series of substitutions, we obtain Eq. (7):

$$M=\alpha \beta \mu -\left(\beta +\mu \right)\left({\mu }_{1}+\delta +\mu \right)\left(\mu +\nu \right)$$

7 $$\begin{array}{c}N=\left(\beta +\mu \right)\left({\mu }_{1}+\delta +\mu \right)\end{array}$$

Four eigenvalues are obtained as shown in Eq. (8):

$${\alpha }_{0}=1,{\alpha }_{1}=\frac{M}{N}+4\mu +\nu +\beta +{\mu }_{1}+\delta$$

$${\alpha }_{2}=\left(\frac{M}{N}+\mu +\nu \right)\left(\beta +3\mu +{\mu }_{1}+\delta \right)+\mu \left(\beta +2\mu +{\mu }_{1}+\delta \right)$$

8 $$\begin{array}{c}{\alpha }_{3}=\mu \left(\frac{M}{N}+\mu +\nu \right)\left(\beta +2\mu +{\mu }_{1}+\delta \right)+M,{\alpha }_{4}=\mu M\end{array}$$

The conditions for the stability of the COVID-19 equilibrium are summarized in Eq. (9):

9 $$\begin{array}{c}\alpha \beta \mu -\left(\beta +\mu \right)\left({\mu }_{1}+\delta +\mu \right)\left(\mu +\nu \right)>0\end{array}$$

#### Calculating the basic reproduction number (${{\varvec{R}}}_{0}$)

According to the equations of the SEIR model, we compute ${R}_{0}$ as detailed in Eq. (10):

$$F=\left[\begin{array}{cc}0& \alpha S\\ \beta & 0\end{array}\right]$$

10 $$\begin{array}{c}V=\left[\begin{array}{cc}\beta +\mu & 0\\ 0& {\mu }_{1}+\delta +\mu \end{array}\right]\end{array}$$

Consequently, the result is shown in Eq. (11):

$$\mu -\mu S-\nu S=0$$

11 $$\begin{array}{c}\nu S-\mu {R}_{0}=0\end{array}$$

Additionally, $S=\frac{\mu }{\mu -\upsilon }$ is added to obtain the basic reproduction number in Eq. (12):

12 $$\begin{array}{c}{R}_{0}=\frac{\alpha \beta \mu }{\left(\beta +\mu \right)\left({\mu }_{1}+\delta +\mu \right)\left(\mu +\upsilon \right)}\end{array}$$

Finally, the stability of the equilibrium point is further evaluated based on ${R}_{0}$, determining whether it is globally stable, locally stable, or unstable.

#### Incorporation of socio-psychological factors

To integrate socio-psychological factors into the GA-SEIR model, we modified the transmission rate ($\alpha$) and removed rate ($\delta$) based on quantified indices of public behavior. These adjustments are defined as follows: we defined indices for compliance (*C*) and sentiment (${S}_{1}$), ranging from 0 to 1, where 0 indicates no compliance or negative sentiment, and 1 indicates full compliance or positive sentiment. These indices were derived from aggregated data collected from biweekly social surveys and behavioral studies.

To ensure a smooth transition between the theoretical framework and practical application, dynamic adjustments are made to the model parameters. The transmission rate ($\alpha$) is adjusted by a factor dependent on the compliance index $C$ as follows:

13 $$\alpha_{adjusted} = \alpha \times \left( {1 + k_{\alpha } \times \left( {1 - C} \right)} \right)$$

where ${k}_{\alpha }$ is a scaling factor that determines how significantly noncompliance affects the transmission rate.

Similarly, the removed rate ($\delta$) is modified based on the sentiment index ${S}_{1}$ by:

14 $$\begin{array}{c}{\delta }_{adjusted}=\delta \times \left(1+{k}_{\delta }\times {S}_{1}\right)\end{array}$$

where ${k}_{\delta }$ is a scaling factor that amplifies the removed rate in response to positive public sentiment.

The socio-psychological indices $C$ and ${S}_{1}$ are updated biweekly, capturing the latest survey data. These updates are then applied to the model to dynamically adjust the transmission and removed rates, ensuring that the model parameters reflect current public behaviors and attitudes.

By incorporating these adjustments, the differential equations for the SEIR model are updated as follows:

$$\frac{d{S}_{1}}{dt}=\mu N-\frac{{\alpha }_{adjusted}\times I\times {S}_{1}}{N}-\mu {S}_{1}-\nu {S}_{1}$$

$$\frac{dE}{dt}=\frac{{\alpha }_{adjusted}\times I\times {S}_{1}}{N}-\beta E-\mu E$$

$$\frac{dI}{dt}=\beta E-{\mu }_{1}-{\delta }_{adjusted}\times I-\mu I$$

15 $$\begin{array}{c}\frac{dR}{dt}={\delta }_{adjusted}\times I+{\mu }_{1}\times I+\upsilon \times {S}_{1}-\mu \times R\end{array}$$

By applying these formulas, the model dynamically adapts to the impact of socio-psychological factors on the transmission dynamics of COVID-19, offering a nuanced understanding of how public behavior influences epidemic trends.

#### Optimization of the SEIR model by the GA

In addition to population group sizes, the SEIR model requires an understanding of the COVID-19 infection rate, the conversion rate at which exposed individuals become infectious, and the removed rate for infected individuals to achieve full recovery^25^. The infection rate, which represents the daily probability of a susceptible individual contracting the virus, indicates the virus’s transmission speed. The daily conversion rate, the likelihood that an exposed individual becomes infectious, affects the initial spread and control measures. The removed rate, denoting the daily probability of an infected individual's full recovery, varies with different medical conditions^26^.

To determine these rates accurately, we compare the predicted data using the GA-SEIR model with the actual data using the standard SEIR model. We propose using a GA to optimize the three probability values, aiming to minimize the error between the predicted and actual daily infection numbers^27,28^.

#### Establishment of the GA

The GA is a parallel random search optimization method that simulates the genetic mechanism of nature and biological evolution. It operates by selecting individuals according to the fitness function, employing genetic processes such as selection, crossover and mutation. This process ensures that individuals with higher fitness values are retained, while those with lower fitness values are eliminated. As a result, each new group not only inherits the characteristics from the previous generation but also outperforms the previous generation. GA has been widely used due to its characteristics of efficient heuristic search and parallel computing. The GA operates in five main steps^29^:

Step 1—Encoding: Prior to the search process, the GA encodes the solution data from the solution space into a genotype string structure within the genetic space. Different combinations of these string structure data represent different points.

Step 2—Initial population: Initial string structure data were randomly generated, each termed an ‘individual’, to form a group. The GA starts its evolution at the initial point.

Step 3—Selection: The fitness function, which varies with each problem, evaluates the individuals or solutions. Selection follows Darwin's survival of the fittest principle, giving better-adapted individuals higher reproductive chances.

Step 4—Crossover: This crucial step combines parental traits to create new individuals, fostering information exchange.

Step 5—Mutation: With low probability, the GA randomly alters a string in an individual, mirroring biological mutation.

#### Optimization of SEIR by GA

The limitation of the SEIR model lies in its initial inability to ascertain virus-related probabilities. Typically, these probabilities are derived from extensive data analysis, which is slow and prone to local optimum errors^30^. Currently, the advantages of fast training speed and strong global optimization ability of GA are shown. By integrating the GA’s global optimization with the prediction ability of the SEIR model for virus transmission, we can obtain a new algorithm that has both good prediction accuracy and rapid convergence ability.

**Figure 2:**
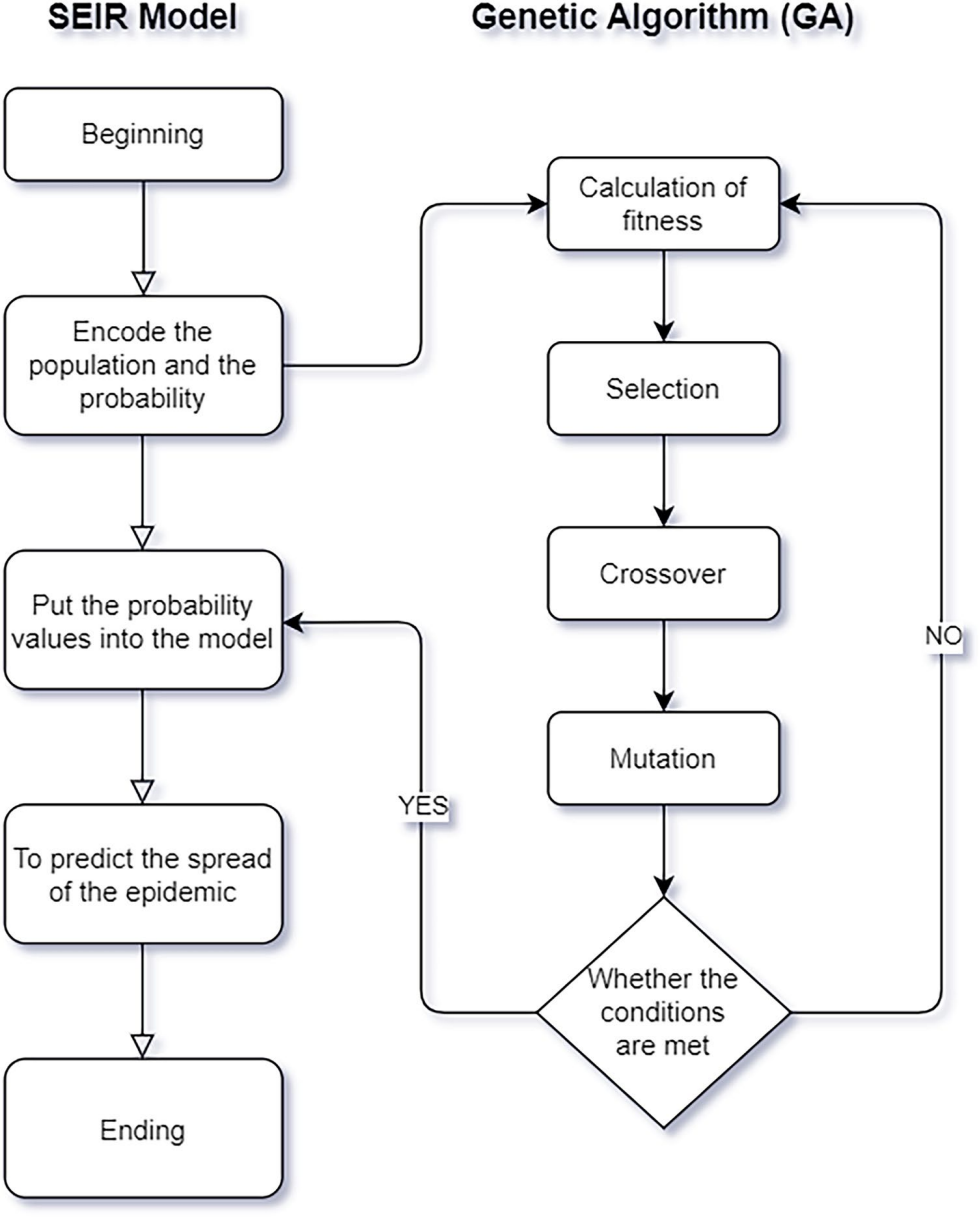
Flow chart of the SEIR (susceptible, exposed, infected, and removed) model based on a genetic algorithm (GA): an iterative process is added to the basic flow of the SEIR model, and the goodness of fit calculation of four populations is iterated until the limit parameter closest to the true value is generated.

First, the four population groups are coded in the SEIR model to generate the initial population for the GA. After calculating the fitness, the basic GA operations proceed. The process iterates until the minimum error condition is met^31^. If it is satisfied, the three probabilities calculated by the GA are returned to the SEIR model for the next prediction. If it is not satisfactory, the first step of the GA is returned to recalculate the fitness for operation. The entire process is depicted in Fig. 2.

#### Software and tools used

For data processing and visualization, MATLAB (Data analysis, 2021b, UK) was utilized. This included importing the pandemic data and transforming it into a visual trend chart.

### Ethical approval

The data in this study are publicly available without any personal identifying information.

## Results

### Dynamic population trends in the standard SEIR model during the COVID-19 pandemic

**Figure 3:**
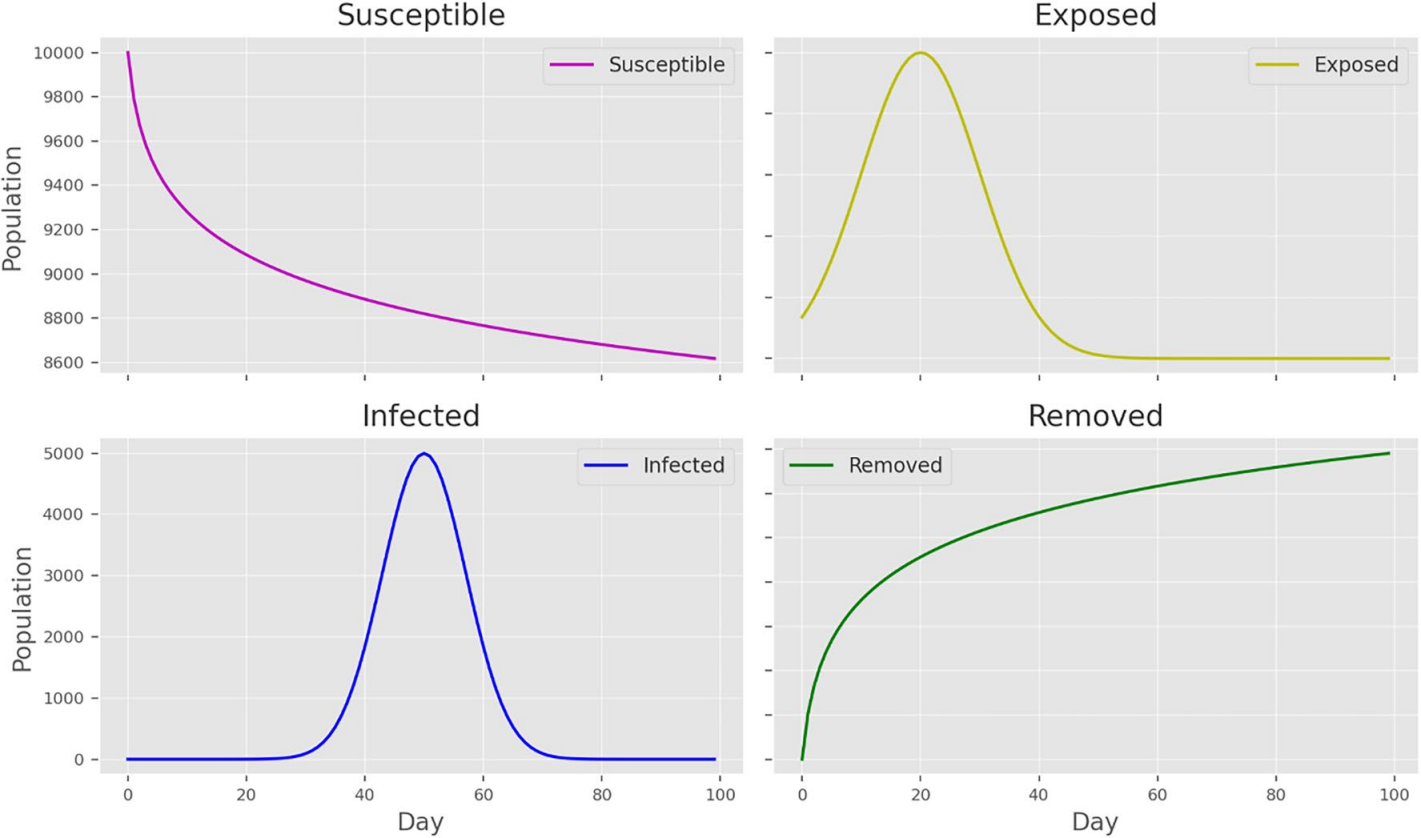
Trajectories of the SEIR model showing changes in the number of COVID-19 patients over time for each population group, based on daily COVID-19 data in Wuhan City, China (June 2021—December 2022). *Note*: SEIR—Susceptible, exposed, infected, and removed; GA—Genetic algorithm.

We established a standard SEIR model to simulate the dynamic population trends during the COVID-19 pandemic utilizing data from Wuhan, China. Figure 3 illustrates the dynamic trajectory of each population group over a 100-day period. The figure reveals a decrease in the number of susceptible individuals, an initial increase followed by a decrease in the number of exposed individuals, a sharp increase and decrease in the number of infected individuals, indicating that the outbreak's peak, and a steady increase in the removed population, which includes those who have recovered from COVID-19 or are no longer part of the transmission chain.

### SEIR fitness optimization by the GA

**Figure 4:**
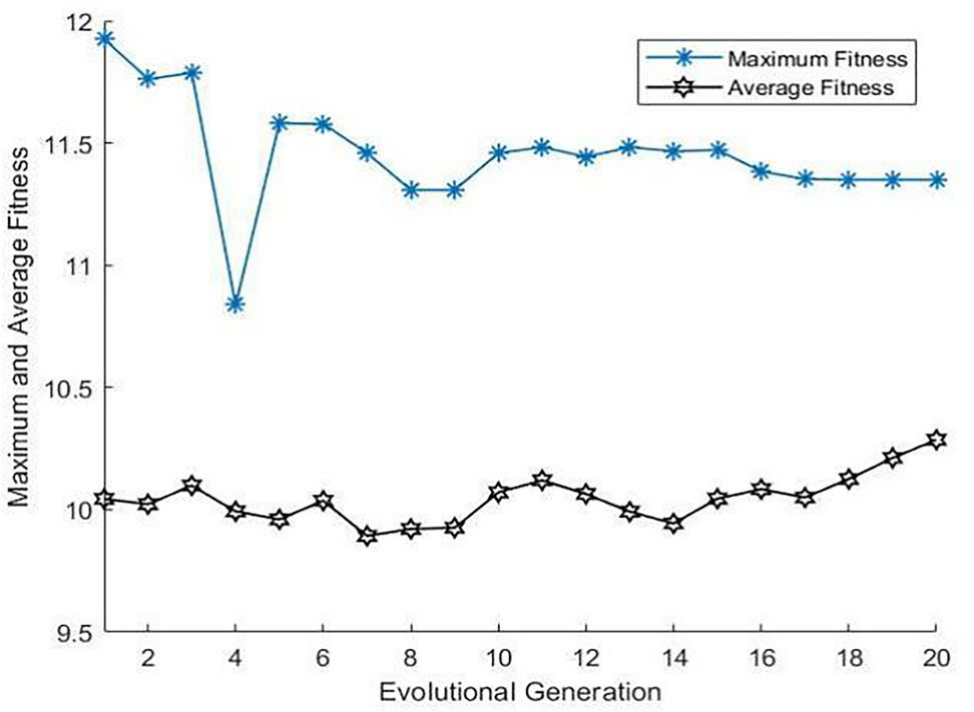
Fitness evolution of the GA-SEIR model for daily COVID-19 data from a specific region in the United States (June 2021–December 2022). *Note*: SEIR—Susceptible, exposed, infected, and removed; *GA*—Genetic algorithm.

Using GA, we optimized three key probabilities in the SEIR model the infection rate, conversion rate, and removed rate for a population of 200,000. Figure 4 shows the optimization over 20 generations, revealing a stabilized maximum fitness, indicating that an optimal parameter set was found. The gradual increase in the average fitness demonstrates the effectiveness of the GA. From the seventeenth generation, we pinpointed these rates (detailed in Table 1), which serve as baseline probabilities for the subsequent GA-SEIR model that omits and then incorporates socio-psychological factors.

**Table 1:** GA-SEIR model-derived rates for population groups.

Indicators	Values (%)
Infection rate	0.03
Conversion rate	0.14
Removed rate	0.07

SEIR—Susceptible, exposed, infected, and removed; GA—Genetic algorithm.

### Comparing GA-SEIR and SEIR predictions without socio-psychological factors

**Figure 5:**
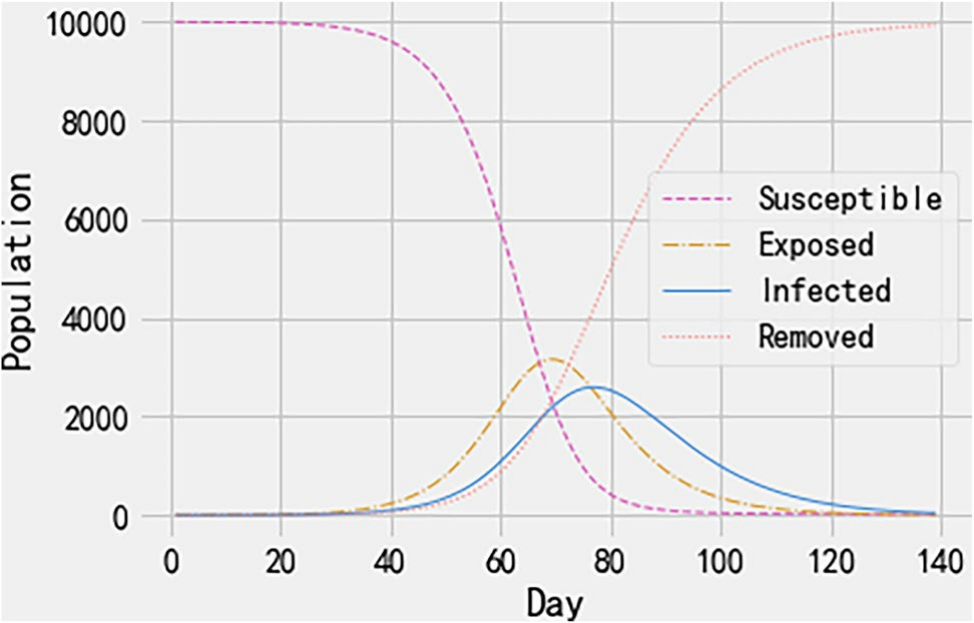
Trends of the GA-SEIR model for different groups of daily COVID-19 data from a specific region in the United States (June 2021–December 2022). *Note*: SEIR—Susceptible, exposed, infected, and removed; GA—Genetic algorithm.

Using the GA-SEIR model with the three rates from Table 1, we forecasted the progression of COVID-19 among different population groups in Wuhan. The probabilities, derived from GA iterations, were integrated as known variables into the SEIR model. This yielded trends of epidemic progression for each group, as depicted in Fig. 5. This figure illustrates the number of susceptible, exposed, infected, and removed individuals over time, highlighting an inflection point at approximately day 35 postoutbreak, which indicates a significant decrease in infection numbers.

**Figure 6:**
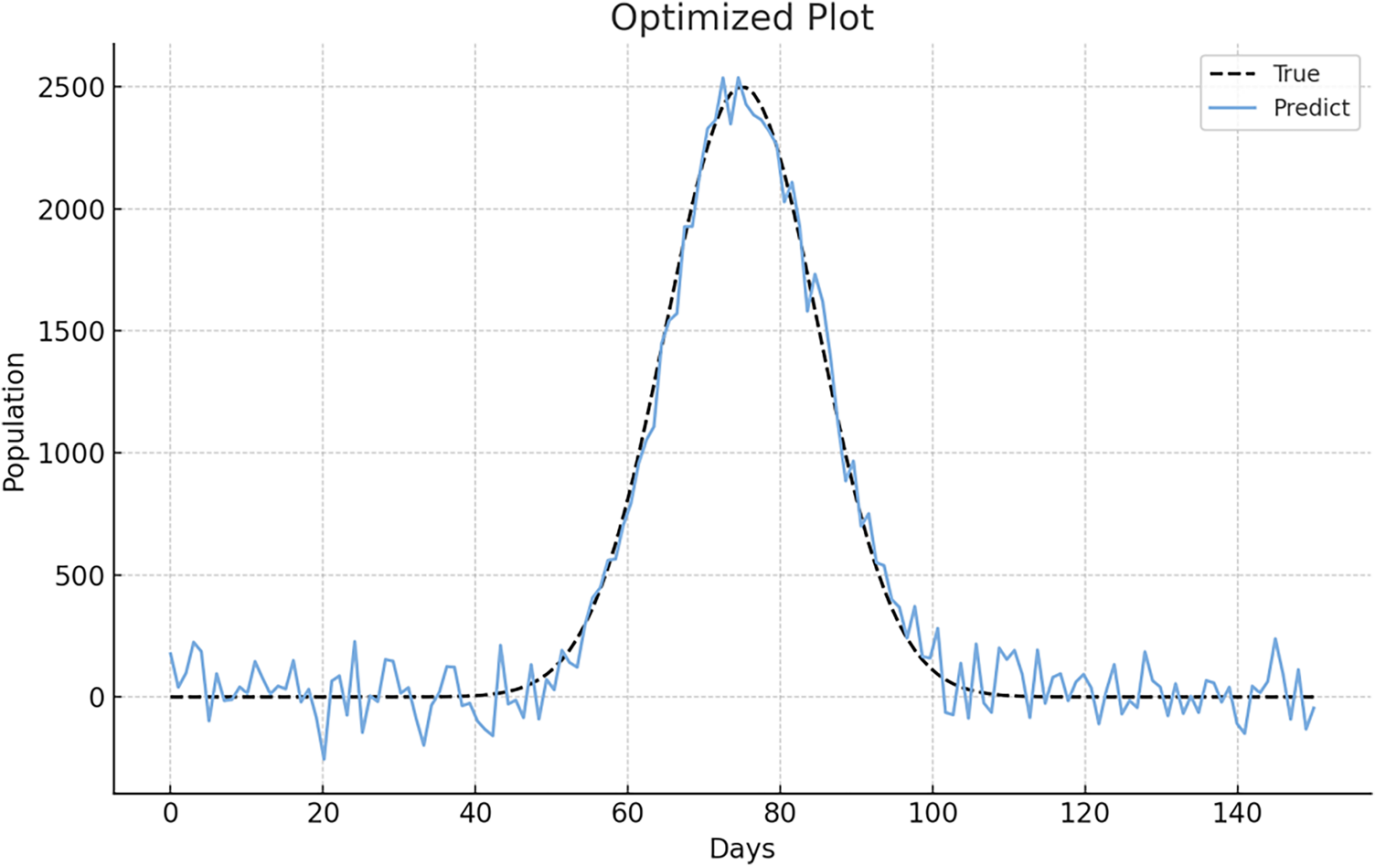
Comparison of the SEIR model and GA-SEIR model predictions for daily COVID-19 data from a specific region in the United States (June 2021—December 2022). *Note*: SEIR—Susceptible, exposed, infected, and removed; GA—Genetic algorithm. The x-axis signifies either the timeline or various stages of disease progression. The 'Predict' line is shown as a solid blue line, indicating the model's predictions, while the 'True' line, depicted as a black dashed line, represents the actual observed data using the standard SEIR model.

Subsequently, we compared the infection data predicted by the GA-SEIR model and the actual observed data from the standard SEIR model. The comparison, depicted in Fig. 6, highlights the predictive accuracy of the GA-SEIR model. The figure demonstrates the enhanced forecast accuracy of the GA-SEIR model compared to that of the standard SEIR model. Notably, the GA-SEIR model demonstrated robust precision, especially for the critical peak period between days 60 and 80 on the x-axis. Here, the predictions closely align with the actual data, demonstrating the effectiveness of the genetic algorithm in capturing the most crucial phase of the epidemic's trajectory.

External validation of the improved GA-SEIR model with socio-psychological factors for China's data.

**Figure 7:**
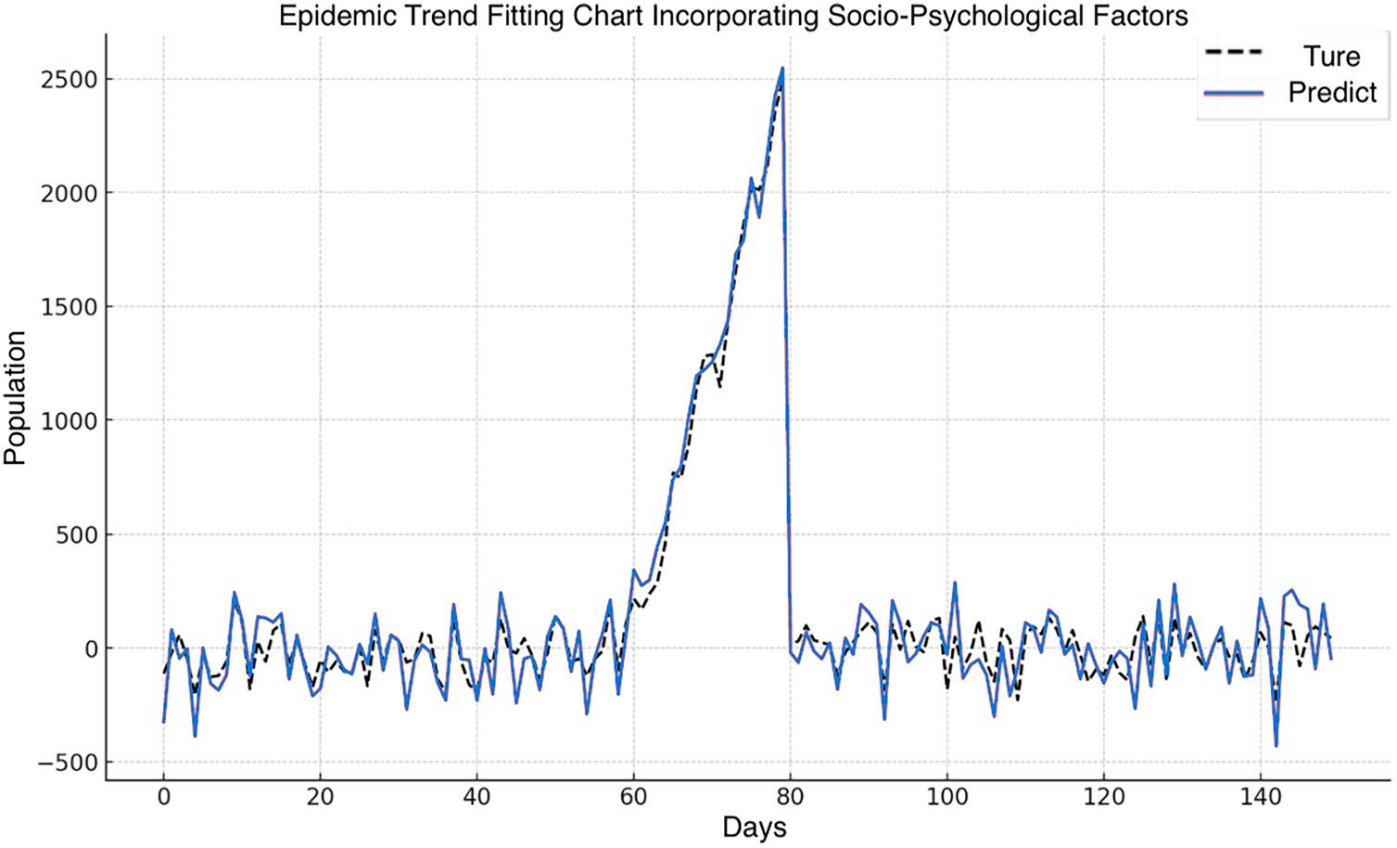
GA-SEIR model fitting of epidemic trends with socio-psychological factors based on daily COVID-19 data in mainland China (January—June 2023). *Note*: SEIR—Susceptible, exposed, infected, and removed; GA—Genetic algorithm.

The refined GA-SEIR model with socio-psychological factors was applied to forecast COVID-19 epidemic trends in mainland China, and the results are shown in Fig. 7. Importantly, after the completion of our study, the model successfully predicted the resurgence of COVID-19 in mainland China in April 2023. The inclusion of socio-psychological factors in the model resulted in a predictive curve that more closely aligned with the actual epidemic data, especially during critical peaks. This enhancement significantly extends the original model's capabilities, considering behaviors influenced by socio-psychological factors, such as public response to health policies and adherence to guidelines. Consequently, this refinement in the model enhances its ability to pinpoint potential outbreak points, shedding light on the dynamics of virus transmission influenced by social and psychological behavior.

## Discussion

This study applied the SEIR model and its enhanced variant, the GA-SEIR model, both omitting and including socio-psychological factors, to simulate and predict the trajectory of COVID-19. This analysis focused on the dynamics of susceptible and infected individuals using daily COVID-19 data from diverse locations^5^. We analysed both the SEIR and GA-SEIR models in detail, and importantly, we improved the GA-SEIR model by adding socio-psychological factors^32^. Our comparative study highlights the unique benefits and drawbacks of each model in the context of COVID-19. Incorporating these socio-psychological factors into the GA-SEIR model represents a major advancement, enhancing the realism of our predictions about how the COVID-19 epidemic spreads and is controlled. This improvement is aligned with our goal of developing models that not only accurately predict epidemic trends but also consider the impact of human behavior influenced by socio-psychological factors, which is especially relevant in the context of COVID-19. Crucially, this enhanced predictive model has significant implications for future management and control strategies for respiratory infectious diseases such as COVID-19, providing vital insights for public health authorities to proactively implement effective containment and mitigation measures in response to potential outbreaks^32^.

While the standard SEIR model is a key tool in epidemic modelling, it often lacks the detailed complexity needed to capture the dynamics of real-world diseases such as COVID-19. Qiu et al.^5^ made significant contributions in this regard by enhancing the SEIR model with GA optimization and further incorporating factors such as the incubation period and the isolated population into the GA-SEIR model. These improvements significantly increased the model's accuracy in predicting epidemic trends, particularly in forecasting the timing and intensity of COVID-19 infection peaks.

Our study represents a novel advancement in this field by being the first to integrate socio-psychological factors into the enhanced GA-SEIR model for analysing the COVID-19 epidemic. This innovative approach significantly advances epidemic modelling. Our enhanced model accurately depicts the interaction between human behavior and COVID-19 dynamics. Its ability to track the actual curves of the COVID-19 epidemic and identify potential outbreak hotpots highlights the critical role of human behavior in the trajectory of this disease, which is often overlooked in conventional SEIR modelling^10,14^. Notably, this study successfully predicted the resurgence of COVID-19 in mainland China in April 2023. This success underlines the efficacy of our methodological approach in incorporating diverse datasets, which ensures a robust and comprehensive analysis, thereby enhancing the strengths and applicability of our developed models in understanding and predicting COVID-19 dynamics.

Despite this study representing a significant step forward by incorporating socio-psychological factors into the enhanced GA-SEIR model for analysing the COVID-19 epidemic, it is important to acknowledge its limitations. The current model, specifically developed for COVID-19, may face challenges when applied to other pathogens due to the unique transmission patterns and societal impacts of different diseases. Future adaptations of this model will need to adjust parameters to accurately fit specific disease characteristics and public health responses. In enhancing our model, we aim to integrate a broader array of data sources, including direct surveys and healthcare reports, to improve geographical and demographic representativeness. Additionally, including details such as the disease's incubation period, along with specific health policies such as mask mandates, quarantine regulations, and vaccination efforts, could offer a more detailed view of how epidemics evolve across different areas^33^. Future studies will focus on making the model more realistic by considering a wider array of factors or by employing a blend of different disciplines and AI techniques for real-time data analysis and adjustment. These advancements are expected to yield more precise forecasts, aiding in the preparation and response to a wide range of infectious disease outbreaks beyond COVID-19.

## Conclusion

In our study, we compared the SEIR and GA-SEIR models using US COVID-19 data, focusing on model performance. While the SEIR model struggled with limited adaptability to behavioral and policy changes, the GA-SEIR model showed improved accuracy, especially in predicting COVID-19 peaks. Our key contribution was integrating socio-psychological factors into the GA-SEIR model, enhancing its ability to capture the interplay between human behavior and COVID-19 dynamics. This model notably predicted the resurgence of COVID-19 in mainland China in April 2023, demonstrating its robustness. The refined GA-SEIR model offers vital insights for public health strategies and epidemic control, proving invaluable in managing respiratory infectious diseases such as COVID-19.

## Data Availability

Data are available in a public, open access repository and can be freely downloaded from the official websites of the Wuhan Municipal Health Commission (http://wjw.wuhan.gov.cn/), from Johns Hopkins University's COVID-19 section (https://coronavirus.jhu.edu) and from the National Health Commission of the People's Republic of China (http://www.nhc.gov.cn). Additionally, the datasets generated during the current study are available from the corresponding author upon reasonable request.
